# Psychometric Evaluation of the Nine-Item Problematic Internet Use Questionnaire (PIUQ-9) in Nine European Samples of Internet Users

**DOI:** 10.3389/fpsyt.2019.00136

**Published:** 2019-03-22

**Authors:** Stéphanie Laconi, Róbert Urbán, Katarzyna Kaliszewska-Czeremska, Daria J. Kuss, Augusto Gnisci, Ida Sergi, Antonia Barke, Franziska Jeromin, Jarosław Groth, Manuel Gamez-Guadix, Neslihan Keser Ozcan, Konstantinos Siomos, Georgios D. Floros, Mark D. Griffiths, Zsolt Demetrovics, Orsolya Király

**Affiliations:** ^1^Laboratoire CERPPS (Centre d'Études et de Recherche en Psychopathologie et Psychologie de la Santé) – EA 7411 – Université Toulouse 2 Jean Jaurès, Toulouse, France; ^2^Institute of Psychology, ELTE Eötvös Loránd University, Budapest, Hungary; ^3^Institute of Psychology, Jesuit University Ignatianum, Krakow, Poland; ^4^International Gaming Research Unit, Nottingham Trent University, Nottingham, United Kingdom; ^5^Department of Psychology, University of Campania “Luigi Vanvitelli”, Caserta, Italy; ^6^Clinical and Biological Psychology, Catholic University Eichstätt-Ingolstadt, Eichstätt, Germany; ^7^Department of Psychology, Philipps University of Marburg, Marburg, Germany; ^8^Institute of Psychology, Adam Mickiewicz University, Poznań, Poland; ^9^Faculty of Psychology, Universidad Autónoma de Madrid, Madrid, Spain; ^10^Department of Midwifery, Faculty of Health Sciences, Istanbul University-Cerrahpasa, Istanbul, Turkey; ^11^Hellenic Association for the Study of Internet Addiction Disorder, Athens, Greece; ^12^Department of Psychiatry, Aristotle University of Thessaloniki, Thessaloniki, Greece

**Keywords:** internet addiction, online addiction, problematic internet use, Problematic Internet Use Questionnaire, screening instrument, psychometric properties, cross-cultural studies

## Abstract

**Objectives:** The nine-item Problematic Internet Use Questionnaire (PIUQ-9) is a brief self-report screening instrument for problematic internet use. The main objective of the present study was to explore the psychometric properties of the PIUQ-9 among nine different language-based samples of European internet users (Italian, German, French, Polish, Turkish, Hungarian, English, and Greek).

**Methods:** The total sample comprised 5,593 internet users (38.1% men), aged between 18 and 87 years (*M* = 25.81; *SD* = 8.61). Via online recruitment, participants completed the PIUQ-9, the Brief Symptom Inventory (BSI) and items about time spent online.

**Results:** Confirmatory factor analysis demonstrated that the bifactor model with one general factor (i.e., general problem) and two-specific factors (i.e., obsession and neglect + control disorder) yielded acceptable or good fit indices in all subsamples except for one. The common variance index in the bifactor model indicated that the general problem factor explained from 57.0 to 76.5% of common variance, which supports the presence of a strong global factor. According to the multiple indicators multiple causes (MIMIC) model, psychiatric symptoms had a moderate-to-strong direct effect on the general problem factor in all subsamples, ranging from β = 0.28 to β = 0.52 supporting the construct validity of the scale. Furthermore, in a majority of the subsamples, time spent online during the weekend had considerably higher effect sizes on the general problem factor than time spent online during weekdays.

**Conclusion:** The present study highlights the appropriate psychometric properties of the PIUQ-9 across a number of European languages and cultures.

## Introduction

Internet addiction or problematic internet use (PIU) have both been defined as an excessive and/or inappropriate use of the internet which can lead to psychological, social, academic, and/or professional difficulties among a small minority of users and which shows high comorbidity with other mental disorders (1–6). PIU has not been consensually recognized as an official disorder and it lacks a consensual definition and agreed upon diagnostic criteria despite the recent introduction of Internet Gaming Disorder in the third section of the (fifth) Diagnostic and Statistical Manual of Mental Disorders [DSM-5; (7)] and Gaming Disorder in the most recent 11th edition of the International Classification of Diseases [ICD-11; (8)]. Nevertheless, many studies have already demonstrated the extent to which PIU is similar to other addictive disorders, such as gambling disorder and psychoactive substance use disorder. Consequently, a significant number of PIU definitions and diagnostic criteria are based on these two disorders (9–12).

PIU has frequently been comorbid with psychopathology, including depressive disorders (13), ADHD (14) and/or other psychiatric disorders and symptomatology (15, 16). Research has also found that psychopathology, operationalized using the Global Severity Index, as well as dysfunctional coping strategies, are predictive of addictive internet use (17).

PIU is typically viewed as an umbrella term referring to several problematic online behaviors (18) (e.g., gaming, gambling, sexual activity, social media use, and shopping), and as such, it has been criticized for being conceptually too heterogeneous [e.g., (19, 20)]. Nevertheless, arguably such umbrella terms can still be useful for screening the general population. Such a screening can identify groups at risk, which can then be explored more closely for the specific behaviors, while avoiding using resources to groups that are not at risk of any problematic online behaviors.

Research into internet addiction has led to the development of numerous assessment tools. Moreover, the use of a large number of different screening instruments led to major inconsistencies in the assessment and prevalence estimations in PIU studies (9, 11). This condition could be improved by using similar methods and screening instruments, as well as tools with appropriate psychometric properties, in order to reliably compare results across studies. However, relatively few validation studies have been conducted (11, 21, 22). In a systematic review published in 2014, Laconi et al., identified 45 measurement instruments for PIU, of which only 17 (38%) had been evaluated more than once in terms of their psychometric properties. Therefore, they concluded that most of the existing scales for PIU require further validation work but some of them have already demonstrated promising psychometric properties relative to other scales, given their solid theoretical basis and/or their rigorous validation studies. In addition, given the global nature of PIU, measurement instruments should also be explored across different cultural and linguistic contexts (5).

The Problematic Internet Use Questionnaire [PIUQ; (23)] is one of the aforementioned screening instruments that has previously demonstrated promising psychometric properties. It was developed to comprehensively assess the main aspects of PIU and several studies have examined its psychometric properties (5, 23, 24). Factorial investigation of the original scale revealed a three-factor solution (5, 23, 24). The three factors identified in the scale were obsession, neglect, and control disorder, reflecting solid content validity. Nevertheless, a French study also explored the validity of a 12-item form and reported a four-factor solution comprising preoccupation, withdrawal, negative outcomes, and self-control (25). Internal consistency of the different forms of the PIUQ has been high in all validating studies, with Cronbach's alphas ranging between 0.77 and 0.91. Its test-retest reliability evaluated in one study was also high (23). Validity was found to be satisfactory with regards to depression [*r*s ranged from 0.20 to 0.43; (21, 24)], time spent online per day [*r* = 0.47; (21)], and anxiety [*r* = 0.73; (25)]. However, there is a need to further test the reliability and validity of the PIUQ in different cultural and linguistic contexts.

Given that the length of the scale is often an important issue in large-scale studies in regard to survey fatigue, brief but comprehensive screening instruments are usually preferred. For this reason, two brief versions have also been proposed for the PIUQ by the original authors including a nine-item (5) and a six-item (21) version, both of which preserved the same three-factor structure as the original 18-item scale. According to the previous validation studies, the PIUQ and its short forms, appear to be promising screening instruments, which are worth exploring further from a psychometric perspective. Based on these outlined concerns and issues, the main objective of the present study was to explore the psychometric properties of the PIUQ 9-item version (still a reasonably comprehensive scale) across nine different language-based samples of European internet users.

## Material and Methods

### Participants and Procedure

All participants were recruited during December 2015 and May 2016 via an online dedicated website which provided information about the study (e.g., objectives and assurances of anonymity and confidentiality). A specific *Facebook* page was also created and was used to recruit participants. Authors of the present paper used their own social networking profiles and the online communication networks of their university or institute to further publicize the study. This recruitment strategy resulted in considerable differences among the samples in terms of gender, age, and other socio-economic variables, as well as differing internet use patterns (see Table 1 in the Results section). More details about the recruitment procedure are presented elsewhere [see (26)]. The present study received the approval from the ethics committee of [masked for review purposes]. Participants who did not give their consent and who were under the age of 18 years were excluded (*n* = 76), as were participants who did not complete the socio-demographic information (*n* = 1,048). For participants who did not complete the PIUQ and the Brief Symptom Inventory (BSI), if <10% of the data were missing, empty cells were replaced by the mean scores of the scale (*n* = 1,249). The completion rate was 70%. Of the total of 7,969 participants, 2,376 participants were excluded. Consequently, the final sample included 5,593 internet users speaking nine languages (Italian, German, French, Polish, Turkish, Hungarian, English, and Greek).

**Table 1:** Descriptive statistics of studied variables.

	**Total sample (*n* = 5,593)**	**Italian *n* = 1,346**	**German *n* = 1,190**	**French *n* = 1,030**	**Polish *n* = 548**	**Spanish *n* = 473**	**Turkish *n* = 432**	**Hungarian *n* = 245**	**English *n* = 175**	**Greek *n* = 154**
Mean age (SD)	25.8 (8.6)	23.6 (5.0)	27.2 (9.5)	25.3 (8.7)	25.4 (7.8)	30.4 (10.7)	23.4 (6.4)	27.1 (9.2)	23.2 (8.8)	32.02 (12.0)
**GENDER**										
Men	2,129 (38.1%)	504 (37.4%)	407 (32.5%)	302 (29.4%)	533 (97.3%)	210 (44.4%)	70 (16.2%)	1 (0.4%)	42 (24.0%)	60 (39.0%)
**STATUS**										
High school students	120 (2.1%)	63 (4.7%)	6 (0.5%)	3 (0.3%)	21 (3.8%)	14 (3%)	3 (0.7%)	8 (3.3%)	–	2 (1.3%)
University students	3,589 (64.2%)	924 (68.6%)	846 (71.1%)	713 (69.2%)	347 (63.3%)	126 (26.6%)	294 (68.1%)	136 (55.5%)	141 (80.6%)	62 (40.3%)
Employed	1,536 (27.5%)	229 (17%)	327 (27.5%)	226 (21.9%)	161 (29.4%)	272 (57.5%)	127 (29.4%)	90 (36.7%)	34 (19.4%)	70 (45.5%)
Not employed	348 (6.2%)	130 (9.7%)	11 (0.9%)	88 (8.5%)	19 (3.5%)	61 (12.9%)	8 (1.9%)	11 (4.5%)	–	20 (13.0%)
**EDUCATIONAL LEVEL**										
In a secondary/high school	76 (1.3%)	13 (0.9%)	2 (0.2%)	6 (0.6%)	23 (4.2%)	13 (2.7%)	3 (0.7%)	8 (3.3%)	4 (2.3%)	4 (2.6%)
Second. school finished (without dipl.)	231 (4.1%)	78 (5.8%)	1 (0.1%)	39 (3.8%)	5 (0.9%)	45 (9.5%)	3 (0.7%)	39 (15.9%)	–	21 (13.6%)
High school diploma	2,768 (49.5%)	867 (64.4%)	718 (60.3%)	407 (39.5%)	295 (53.8%	143 (30.2%)	52 (12%)	115 (46.9%)	127 (72.6%)	44 (28.6%)
BA degree/engineer dipl.	1,486 (26.6%)	297 (22.1%)	175 (14.7%)	354 (34.4%)	61 (11.1%)	167 (35.3%)	317 (73.4%)	54 (22%)	27 (15.4%)	34 (22.1%)
MA diploma	843 (15.1%)	75 (5.6%)	227 (19.1%)	199 (19.3%)	141 (25.7%)	95 (20.1%)	38 (8.8%)	23 (9.4%)	11 (6.3%)	34 (22.1%)
PhD/doctorate	189 (3.4%)	16 (1.2%)	67 (5.6%)	25 (2.4%)	23 (4.2%)	10 (2.1%)	19 (4.4%)	6 (2.4%)	6 (3.4%)	17 (11.0%)
PIUQ-9 mean scores (SD)	17.8 (6.2)	17.9 (6.2)	16.2 (5.1)	17.9 (5.9)	18.1 (6.3)	17.2 (6.3)	19.4 (7.6)	17.9 (6.3)	22.2 (6.4)	19.8 (7.1)
Time online—Week (from Monday to Friday in hours), mean (SD)	8.9 (12.0	11.6 (15.2)	5.6 (7.8)	8.9 (11.0)	5.1 (4.1)	4.2 (3.5)	18.8 (15.7)	5.4 (6.6)	15.1 (14.2)	8.5 (11.0)
Time online—Weekend (Saturday and Sunday in hours), mean (SD)	5.9 (5.2)	5.9 (5.5)	5.1 (4.6)	6.7 (5.6)	5.4 (3.3)	4.0 (3.5)	7.9 (6.5)	5.5 (4.0)	8.6 (6.9)	5.5 (5.1)
BSI mean score (SD)	0.6 (0.5)	0.6 (0.5)	0.4 (0.4)	0.5 (0.5)	0.7 (0.6)	0.4 (0.5)	0.8 (0.7)	0.7 (0.6)	0.7 (0.6)	0.5 (0.6)

*SD, Standard deviation; PIUQ, Problematic Internet Use Questionnaire; BSI, Brief Symptom Inventory*.

### Measures

Basic sociodemographic information (gender, age, education, working status, the size of the residence) along with information regarding internet use were collected. Using an open question, time spent online was assessed separately for the weekdays (from Monday to Friday) and for the weekend days (Saturday and Sunday) in hours per day.

Problematic internet use was assessed using the nine-item version of the Problematic Internet Use Questionnaire [PIUQ-9; (5); see all nine language versions in the Appendix]. The original scale comprised 18 items consisting of three subscales: obsession, neglect, and control disorder (23). During the development of the short nine-item version, the authors aimed to retain the original three-factor structure assessed by three items, respectively. A 5-point Likert scale (ranging from “never” to “always/almost always”) was used to estimate how much the given statements characterized the respondents. Scores range from 9 to 45, with higher scores indicating higher risk of PIU. In the present study, internal consistency of the PIUQ-9 ranged from 0.81 (German subsample) to 0.90 (Turkish subsample).

Psychiatric symptoms were assessed using the Brief Symptom Inventory [BSI; (27)], an instrument that assesses self-reported clinically relevant psychological symptoms. The 53-item questionnaire uses a 5-point Likert scale (from 0 = “not at all” to 4 = “extremely”). The BSI comprises nine symptom dimensions: somatization, obsession compulsion, interpersonal sensitivity, depression, anxiety, hostility, phobic anxiety, paranoid ideation, and psychoticism. However, in the present study, the summarized measure—the Global Severity Index (GSI)—was applied to capture the intensity of general psychiatric distress. In the present study, internal consistency of the BSI ranged from 0.95 (German subsample) to 0.98 (Turkish subsample).

Survey questions were translated from English to the other languages using the double back-translation procedure (28). Translation was conducted by research colleagues from the collaborating countries and back translated by another expert. Back translations were then compared with the original English version and differences were discussed until a final consensus was reached.

### Statistical Analysis

Confirmatory factor analysis (CFA) with maximum likelihood estimation with robust standard errors (MLR) in Mplus 8 (29) was used to estimate the degree of fit of the previously proposed three-factor model and three alternative models in all language-based subsamples. Alternative models were tested because the original three-factor model did not yield acceptable fit in all subsamples, and two of the three factors (neglect and control disorder) were highly correlated with each other. The three alternative models were the following: (i) a two-factor model where neglect and control disorder factors were merged; (ii) a bifactor model representing a global severity dimension on which each item is loaded plus three specific factors on which the items belonging to the three original factors (obsession, neglect and control disorder) were loaded (and where the correlations between specific factors were fixed at zero); (iii) a bifactor model representing a global severity dimension on which each item was loaded plus two specific factors on which the items belonging to the two newly created factors (obsession and neglect + control disorder) were loaded (where the correlations between specific factors were also fixed at zero). Missing data were treated with the full information maximum likelihood estimation method (29). The following fit indices were used in the CFA analysis: chi-square test (χ^2^), comparative fit index (CFI), Tucker-Lewis index (TLI), root mean square error of approximation (RMSEA) and its pclose value, and standardized root mean residual (SRMR). According to Brown and Moore (30), in practice, it is suggested that researchers report and consider each of the aforementioned fit indices because they provide different types of information [see (31) for details] and considered together, they yield a more conservative and reliable evaluation of model fit. The model can be considered satisfactory when the CFI and TLI values are higher than or close to 0.95 and still acceptable if the values are above 0.90 (31). An RMSEA value of below 0.05 indicates excellent fit, a value around 0.08 indicates adequate fit, and a value above 0.10 indicates poor fit. The pclose value is a statistical test, which evaluates the statistical deviation of the RMSEA value from 0.05, therefore non-significant pclose values (*p* > 0.05) indicate good model fit (31). SRMR values below 0.10 indicate a good fit. Sample size adjusted Bayesian Information Criteria (SSABIC) was also calculated to compare the competing models; lower values indicate models that are more parsimonious. Because the models are based on the same set of observed variables, sample size adjusted Bayesian Information Criteria index was used in model selection beside the parsimony and interpretability of the models.

In order to quantify the degree of unidimensionality in bifactor models, the percentage of common variance attributable to the general factor through the use of explained common variance (ECV) index (32, 33) was applied. Additionally, omega and omega hierarchical indices were used to measure how precisely a self-reported symptom scale score assesses the combination of general and specific constructs and a certain target construct (34). In the evaluation of the specific factors, we also used an index of construct replicability, or H index (35), which provides the correlation between a factor and an optimally weighted item composite (36). When H is low, the latent variable is not well-defined by the indicators and, thus, is expected to change across studies, whereas when H is high (>0.70), the latent variable is well-defined by its indicators and shows stability across studies (36).

The present study also tested measurement invariance across languages using multiple-group CFAs with a convenience feature of Mplus in one model (omnibus test of invariance) (29). In the first step, models with increasingly constrained parameters were estimated: (i) factor loadings and intercepts were freely estimated across groups (configural invariance), (ii) factor loadings were set to be equal across groups (metric invariance), and (iii) factor loadings and intercepts were set to be equal across groups (scalar invariance). When comparing the increasingly constrained models, due to the oversensitivity of the chi-square difference test (37), relative change in fit indices (i.e., ΔCFI and ΔRMSEA) were also examined. A change of ≥ −0.01 in the CFI and a change of ≥0.015 in the RMSEA indicates non-invariance (38, 39).

Finally, CFA with covariates or multiple indicators multiple causes (MIMIC) confirmatory factor analysis was carried out in all language-based subsamples to test the construct validity of the best fitting structure (the bifactor model with two specific factors) by exploring the associations between the general and specific factors and three possible predictors (i.e., psychiatric symptoms, time spent online on weekdays, and time spent online during weekend days). CFA with covariates or MIMIC can estimate the effect of indicators on latent variables at the same time when direct effects of grouping variables or other continuous variables on the latent variables are also included. The “outcome” variables of the model were the latent constructs of the bifactor model (i.e., the general factor and the two specific factors). The model was then complemented with the structural part by including a set of exogenous variables, in this case psychiatric symptoms and time spent using the internet during weekdays and weekends to investigate the effects of these variables (“causes”) on the latent constructs. Gender and age were introduced in all models as control variables.

## Results

### Descriptive Statistics

Descriptive statistics of the total sample and the nine subsamples are detailed in Table 1 (i.e., mean age, gender, status, educational level, mean scores of PIU, time spent online and psychopathology). The mean age of the total sample was approximately 25.8 years and varied between 23.3 years (English sample) and 32.0 years (Greek sample) for the subsamples. Approximately two-fifths of the total sample was male (38.1%). However, the subsamples differed substantially regarding gender distribution. For instance, the Hungarian sample had one single male respondent, while only 3% of the Polish sample was female. The majority of the participants were university students (64.2% of the total sample), except in the Spanish and the Greek samples, where there were more employed participants than students (57.5 and 45.5%, respectively). Time spent online also varied considerably across the samples with Turkish- and English-speaking participants spending the most time online both during the week and the weekends.

### Measurement Models for the PIUQ-9

We tested four measurement models across all languages. The fit indices are reported in Table 2. The χ^2^ test was significant in all four models across all languages. However, this test is oversensitive to large sample size. Therefore, the other fit indices (i.e., CFI, TLI, RMSEA, and SRMR) were compared in all subsamples. The original three-factor model yielded acceptable fit indices in four of the nine subsamples (Italian, German, Turkish, and Greek). The two-factor model in which the neglect and control disorder factors were merged because of the high correlation between them produced acceptable fit in two of the nine subsamples (Turkish and Greek). The bifactor model with the three specific factors had acceptable fit indices in six out of nine languages (Italian, German, Spanish, Turkish, English, and Greek). Finally, the bifactor model with two specific factors yielded acceptable or good fit indices in all subsamples except the Hungarian one. In this latter subsample, the TLI and RMSEA indices were those that did not meet the suggested thresholds. However, this model had considerably better fit than the other three models in all subsamples including the Hungarian one, where it was close to an acceptable fit. Furthermore, this model was the most parsimonious according to the SSABIC values. Consequently, the bifactor model with two specific factors was chosen as the best fitting model across the nine languages.

**Table 2:** Confirmatory factor analysis of four measurement models of PIUQ-9.

	**χ^**2**^**	**df**	***p***	**CFI**	**TLI**	**RMSEA**	**RMSEA pclose**	**SRMR**	**SSABIC**
**ITALIAN VERSION (*****n*** **=** **1,346)**									
3-factor model	192.2	24	<0.001	0.941	0.912	0.072 [0.063–0.082]	<0.001	0.050	
2-factor model	245.7	26	<0.001	0.923	0.893	0.079 [0.070–0.088]	<0.001	0.053	
Bifactor model with 3 specific factors^c^	116.4	19	<0.001	0.966	0.935	0.062 [0.051–0.073]	0.034	0.031	30801.9
Bifactor model with 2 specific factors^f^	103.7	19	<0.001	0.970	0.944	0.058 [0.047–0.069]	0.117	0.029	30782.5
**GERMAN VERSION (*****n*** **=** **1,190)**									
3-factor model	152.2	24	<0.001	0.941	0.911	0.067 [0.057–0.077]	0.003	0.050	
2-factor model	225.5	26	<0.001	0.908	0.873	0.080 [0.071–0.090]	<0.001	0.058	
Bifactor model with 3 specific factors^d^	58.5	19	<0.001	0.982	0.966	0.042 [0.030–0.054]	0.856	0.025	24887.3
Bifactor model with 2 specific factors	49.3	18	<0.001	0.986	0.971	0.038 [0.026–0.051]	0.932	0.018	24864.7
**FRENCH VERSION (*****n*** **=** **1,030)**									
3-factor model	245.4	24	<0.001	0.893	0.839	0.095 [0.084–0.106]	<0.001	0.059	
2-factor model	258.9	26	<0.001	0.887	0.844	0.093 [0.083–0.104]	<0.001	0.059	
Bifactor model with 3 specific factors	139.8	18	<0.001	0.941	0.882	0.081 [0.069–0.094]	<0.001	0.039	23986.1
Bifactor model with 2 specific factors	98.2	18	<0.001	0.961	0.922	0.066 [0.053–0.079]	0.019	0.031	23935.0
**POLISH VERSION (*****n*** **=** **548)**									
3-factor model	149.7	24	<0.001	0.909	0.864	0.098 [0.083–0.113]	<0.001	0.058	
2-factor model	154.7	26	<0.001	0.907	0.871	0.095 [0.081–0.110]	<0.001	0.058	
Bifactor model with 3 specific factors^c^	103.6	19	<0.001	0.939	0.884	0.090 [0.074–0.107]	<0.001	0.042	12530.5
Bifactor model with 2 specific factors	68.7	18	<0.001	0.963	0.927	0.072 [0.054–0.090]	0.022	0.030	12488.1
**SPANISH VERSION (*****n*** **=** **473)**									
3-factor model*	104.1	24	<0.001	0.923	0.885	0.084 [0.068–0.101]	<0.001	0.051	
2-factor model	111.7	26	<0.001	0.918	0.886	0.083 [0.068–0.100]	<0.001	0.052	
Bifactor model with 3 specific factors^e^	65.9	19	<0.001	0.955	0.915	0.072 [0.054–0.092]	0.026	0.038	10599.9
Bifactor model with 2.specific factors	47.3	18	<0.001	0.972	0.944	0.059 [0.039–0.079]	0.221	0.030	10566.0
**TURKISH VERSION (*****n*** **=** **432)**									
3-factor model	70.8	24	<0.001	0.964	0.946	0.067 [0.049–0.086]	0.056	0.037	
2-factor model	73.3	26	<0.001	0.964	0.950	0.065 [0.048–0.083]	0.076	0.036	
Bifactor model with 3 specific factors^c^	42.7	19	0.001	0.982	0.965	0.054 [0.032–0.075]	0.359	0.027	10171.0
Bifactor model with 2 specific factors^b^	25.9	19	0.133	0.995	0.990	0.029 [0.000–0.054]	0.905	0.018	10147.9
**HUNGARIAN VERSION (*****n*** **=** **245)**									
3-factor model	97.4	24	<0.001	0.884	0.826	0.112 [0.089–0.135]	<0.001	0.066	
2-factor model	107.1	26	<0.001	0.872	0.822	0.113 [0.091–0.135]	<0.001	0.066	
Bifactor model with 3 specific factors	70.2	18	<0.001	0.917	0.835	0.109 [0.083–0.136]	<0.001	0.044	5618.7
Bifactor model with 2 specific factors^b^	57.0	19	<0.001	0.940	0.886	0.090 [0.064–0.118]	0.008	0.035	5604.2
**ENGLISH VERSION (*****n*** **=** **175)**									
3-factor model	51.4	24	<0.001	0.931	0.897	0.081 [0.050–0.111]	0.050	0.062	
2-factor model	96.8	26	<0.001	0.822	0.753	0.125 [0.099–0.152]	<0.001	0.079	
Bifactor model with 3 specific factors^e^	38.7	19	0.005	0.950	0.906	0.077 [0.041–0.112]	0.097	0.048	4360.4
Bifactor model with 2 specific factors^b^	24.5	19	0.176	0.986	0.974	0.041 [0.000–0.082]	0.595	0.036	4346.4
**GREEK VERSION (*****n*** **=** **154)**									
3-factor model^a^	43.5	24	0.009	0.963	0.944	0.073 [0.036–0.107]	0.134	0.042	
2-factor model	45.2	26	0.011	0.964	0.950	0.069 [0.033–0.102]	0.166	0.044	
Bifactor model with 3 specific factors^b^^,^^e^	29.5	20	0.078	0.982	0.967	0.056 [0.000–0.096]	0.379	0.031	3636.9
Bifactor model with 2 specific factors^b^	25.0	19	0.160	0.989	0.978	0.045 [0.000–0.089]	0.524	0.028	3634.4

*PIUQ-9, Problematic Internet Use Questionnaire−9 items; CFI, comparative fit index; TLI, Tucker-Lewis Index; RMSEA, root-mean-square error of approximation; SRMR, standardized root mean residual; SSABIC, sample-size adjusted Bayesian Information Criteria*.

a *Error message (the model is instable due to a correlation larger than 1 between Control disorder and Neglect factors)*.

b *Residual variance of item 1 is fixed to zero*.

c *Residual variance of item 4 is fixed to zero*.

d *Residual variance of item 8 is fixed to zero*.

e *Residual variance of item 5 is fixed to zero*.

f *Residual variance of item 6 is fixed to zero*.

Factor loadings for the bifactor model with two specific factors across all languages are presented in Table 3. In this model, all items loaded significantly on the global PIU factor across all languages. The factor loadings ranged from 0.19 (Item 1 in the English subsample) to 0.77 (Item 4 in the Turkish subsample). Regarding the first specific factor (obsession), Item 9 had non-significant or low loadings on its specific factor, while having strong significant loading on the main factor (the general problem factor) across all subsamples. Regarding the second specific factor that merged two of the original factors (neglect and control disorder), Items 5, 7, and 8 had non-significant or low loadings on their specific factor, while also having strong significant loadings on the general problem factor across all languages.

**Table 3:** Standardized factor loadings of the bifactor model with two specific factors of PIUQ-9.

	**General factor**	**Specific factors**	
		**Obsession**	**Neglect + control disorder**
**ITALIAN VERSION (*****n*** **=** **1,346)**			
Item 3	0.700	0.330	
Item 6	0.598	0.801	
Item 9	0.644	−0.006^ns^	
Item 5	0.521		0.134
Item 8	0.567		0.046^ns^
Item 2	0.562		0.279
Item 4	0.677		0.462
Item 7	0.571		0.146
Item 1	0.449		0.636
**GERMAN VERSION (*****n*** **=** **1,190)**			
Item 3	0.594	0.537	
Item 6	0.385	0.635	
Item 9	0.597	0.255	
Item 5	0.524		0.161 ^ns^
Item 8	0.501		0.099
Item 2	0.574		0.259
Item 4	0.563		0.540
Item 7	0.573		0.183^ns^
Item 1	0.353		0.831
**FRENCH VERSION (*****n*** **=** **1,030)**			
Item 3	0.565	0.578	
Item 6	0.433	0.664	
Item 9	0.686	0.212	
Item 5	0.475		0.208
Item 8	0.600		0.104
Item 2	0.503		0.379
Item 4	0.582		0.452
Item 7	0.648		0.089^ns^
Item 1	0.268		0.731
**POLISH VERSION (*****n*** **=** **548)**			
Item 3	0.674	0.402	
Item 6	0.544	0.683	
Item 9	0.725	0.199^ns^	
Item 5	0.519		0.160^ns^
Item 8	0.614		0.125^ns^
Item 2	0.583		0.494
Item 4	0.606		0.456
Item 7	0.665		0.182
Item 1	0.338		0.728
**SPANISH VERSION (*****n*** **=** **473)**			
Item 3	0.668	0.709^ns^	
Item 6	0.643	0.343^ns^	
Item 9	0.740	0.085^ns^	
Item 5	0.649		0.108^ns^
Item 8	0.551		0.179^ns^
Item 2	0.521		0.415
Item 4	0.609		0.503
Item 7	0.751		0.062^ns^
Item 1	0.382		0.629
**TURKISH VERSION (*****n*** **=** **432)**			
Item 3	0.759	0.261	
Item 6	0.746	0.278	
Item 9	0.748	0.491	
Item 5	0.704		−0.029^ns^
Item 8	0.699		0.097^ns^
Item 2	0.695		0.171
Item 4	0.771		0.261
Item 7	0.676		0.067^ns^
Item 1	0.397		0.918
**HUNGARIAN VERSION (*****n*** **=** **245)**			
Item 3	0.614	0.519	
Item 6	0.525	0.668	
Item 9	0.624	0.288	
Item 5	0.650		0.124^ns^
Item 8	0.625		0.119^ns^
Item 2	0.615		0.285
Item 4	0.548		0.481
Item 7	0.643		0.188
Item 1	0.361		0.933
**ENGLISH VERSION (*****n*** **=** **175)**			
Item 3	0.705	0.236^ns^	
Item 6	0.592	0.523^ns^	
Item 9	0.679	0.170^ns^	
Item 5	0.646		0.039^ns^
Item 8	0.581		0.091^ns^
Item 2	0.571		0.267
Item 4	0.535		0.522
Item 7	0.417		0.306
Item 1	0.192		0.981
**GREEK VERSION (*****n*** **=** **154)**			
Item 3	0.663	0.371^ns^	
Item 6	0.605	0.774^ns^	
Item 9	0.631	0.321^ns^	
Item 5	0.564		0.023^ns^
Item 8	0.634		0.148^ns^
Item 2	0.635		0.220
Item 4	0.757		0.320
Item 7	0.736		0.159
Item 1	0.516		0.857

*PIUQ-9, Problematic Internet Use Questionnaire-−9 items version. All loadings were significant at p < 0.05, except when mentioned. ns, not significant*.

The explained common variance (ECV) index was estimated in the bifactor model and it was found that regarding the PIUQ-9, the general problem factor explained from 57.0% (German subsample) to 76.5% (Turkish subsample) of common variance, which supports the presence of a strong global factor. The explained common variances of specific factors are also reported in Table 4. These varied from 6.8% (Turkish subsample) to 18.6% (French subsample) in the case of the obsession subscale and from 16.7% (Turkish subsample) to 30.3% (English subsample) in the case of the second specific factor (neglect + control disorder). We also calculated omega and omega hierarchical indices to denote how precisely a self-reported symptom scale score assesses the combination of general and specific constructs, and a specific target construct. To evaluate the measurement precision of each subscale in assessing the blend of global PIU and specific factors we calculated coefficient omega; and in assessing only specific problems or only global PIU we computed coefficient omega hierarchical [for details, see (34)]. All omega and omega hierarchical coefficients are reported in Table 3. Omega coefficients varied between 76.5% (German subsample) to 95.4% (Greek subsample) in the case of the obsession specific factor and between 79.7% (French subsample) and 92.0% (Greek subsample) for the merged specific factor. In the case of the omega hierarchical coefficients, coefficients varied between 14.1% (English subsample) and 36.7% (Greek subsample) for the obsession specific factor and between 11.4% (Turkish subsample) and 29.4% (English subsample) in the case of the merged specific factor. Although there is no clear cut-off for the omega hierarchical coefficients, the present authors argue that these coefficients are salient because the specific factors explained approximately 10-35% of the PIU score. Finally, the neglect + control disorder factor showed good construct replicability because its H value was above the suggested criterion (>0.70) in seven samples, and in two additional samples these values were close to 0.70. The obsession factor, on the other hand, was characterized by weak replicability, with its value being larger than 0.70 in only two samples.

**Table 4:** Indicators of dimensionality and reliability of the bifactor model of PIUQ-9.

	**General factor**	**Specific factors**	
	**General problem**	**Obsession**	**Neglect + control disorder**
**ITALIAN VERSION**			
ECV	0.677	0.131	0.191
Ω	0.884	0.797	0.827
Ωh	0.782	0.226	0.134
H	0.840	0.489	0.788
**GERMAN VERSION**			
ECV	0.570	0.174	0.257
Ω	0.858	0.765	0.809
Ωh	0.665	0.345	0.251
H	0.781	0.535	0.736
**FRENCH VERSION**			
ECV	0.600	0.186	0.214
Ω	0.862	0.805	0.797
Ωh	0.682	0.344	0.231
H	0.804	0.572	0.621
**POLISH VERSION**			
ECV	0.649	0.136	0.215
Ω	0.893	0.837	0.838
Ωh	0.729	0.254	0.246
H	0.843	0.526	0.641
**SPANISH VERSION**			
ECV	0.700	0.126	0.174
Ω	0.898	0.851	0.836
Ωh	0.773	0.200	0.193
H	0.863	0.535	0.556
**TURKISH VERSION**			
ECV	0.765	0.068	0.167
Ω	0.927	0.869	0.882
Ωh	0.852	0.150	0.114
H	0.902	0.322	0.846
**HUNGARIAN VERSION**			
ECV	0.601	0.156	0.244
Ω	0.897	0.820	0.858
Ωh	0.719	0.338	0.238
H	0.830	0.558	0.878
**ENGLISH VERSION**			
ECV	0.620	0.077	0.303
Ω	0.873	0.781	0.818
Ωh	0.706	0.141	0.294
H	0.826	0.318	0.963
**GREEK VERSION**			
ECV	0.658	0.168	0.174
Ω	0.955	0.954	0.920
Ωh	0.817	0.367	0.168
H	0.912	0.859	0.938

*PIUQ-9, Problematic Internet Use Questionnaire 9-items version; ECV, explained common variance; Ω, omega; Ωh, omega hierarchical; H, H index*.

Measurement invariance of the bifactor model with two specific factors across all languages were also tested. The fit indices of the configural (equal factor structure), metric (equal factor loadings), and scalar (equal factor loadings and equal intercepts) invariance are reported in Table 5. Configural invariance was supported, however, metric and scalar invariance were not. Imposing the equality constrains (metric invariance) resulted in decreasing degree of fit (Δχ^2^ = 284.3 Δdf = 120 *p* < 0.001; ΔRMSEA = 0.004, ΔCFI = 0.013, ΔSRMR = 0.024). Comparison of scalar against metric invariance also resulted in decreasing degree of fit (Δχ^2^ = 1152.3 Δdf = 48, *p* < 0.001; ΔRMSEA = 0.028, ΔCFI = 0.065, ΔSRMR = 0.020).

**Table 5:** Goodness-of-fit statistics and information criteria for the estimated models relating to the PIUQ-9.

**Measurement models**	**MLR **χ^2^** (*df*)**	**CFI**	**TLI**	**RMSEA [90% CI]**	**SRMR**	**Comparison**	**Δ*χ*^2^ (*df*)**	**ΔCFI**	**ΔTLI**	**ΔRMSEA**	**ΔSRMR**
Configural model (M1)	481.4^*^ (162)	0.975	0.949	0.056 [0.051–0.062]	0.028	–	–	–	–	–	
Metric model (M2)	761.5^*^ (282)	0.962	0.956	0.052 [0.048–0.057]	0.052	M2 vs. M1	284.3^*^ (120)	−0.013	−0.007	−0.004	0.024
Scalar model (M3)	1636.3^*^ (330)	0.897	0.899	0.080 [0.076–0.084]	0.072	M3 vs. M2	1152.3^*^ (48)	−0.065	−0.057	0.028	0.020

*PIUQ-9, Problematic Internet Use Questionnaire 9-item version; MLR, maximum likelihood estimation with robust standard errors; χ^2^, Chi-square; df, degrees of freedom; CFI, comparative fit index; TLI, Tucker-Lewis Index; RMSEA, root-mean-square error of approximation; 90% CI, 90% confidence interval of the RMSEA; SRMR, standardized root mean residual; Δχ^2^, Chi-square difference test; ΔCFI, change CFI value; ΔRMSEA, change in RMSEA value; ΔSRMR, change in SRMR value*.

* *p < 0.001*.

### Multiple Indicator Multiple Cause (MIMIC) Model

In the second step, the structural part of the model was added to the best fitting measurement model (i.e., the bifactor model with two specific factors) and the MIMIC model was estimated in all language-based subsamples. The analysis tested the construct validity of the bifactor model by exploring the associations between the general and specific factors and three possible predictors, namely, psychiatric symptoms and time spent using the internet during weekdays and weekends. Gender and age were introduced in all models as control variables (see Table 6).

**Table 6:** MIMIC model with standardized coefficients (gender and age were both controlled for in the models).

	**General problem**	**Obsession**	**Neglect + Control disorder**
**ITALIAN VERSION (*****n*** **=** **1,328)**			
Psychiatric symptoms	0.51^***^	−0.04	−0.04
Time spent online (weekdays)	−0.14^**^	−0.03	0.10
Time spent online (weekend)	0.31^***^	−0.02	−0.15
*R^2^*	0.34^***^	0.05^*^	0.06
**GERMAN VERSION (*****n*** **=** **1,187)**			
Psychiatric symptoms	0.28^***^	0.15^**^	0.09^*^
Time spent online (weekdays)	0.05	0.00	0.03
Time spent online (weekend)	0.07	0.03	−0.05
*R^2^*	0.13^***^	0.07^**^	0.07^***^
**FRENCH VERSION (*****n*** **=** **1,030)**			
Psychiatric symptoms	0.42^***^	0.11	0.13^*^
Time spent online (weekdays)	−0.10	0.02	0.00
Time spent online (weekend)	0.22^***^	0.07	−0.04
*R^2^*	0.26^***^	0.07^**^	0.07^**^
**POLISH VERSION (*****n*** **=** **548)**			
Psychiatric symptoms	0.38^***^	0.13^*^	0.15^*^
Time spent online (weekdays)	0.07	0.05	0.05
Time spent online (weekend)	0.20^***^	0.00	0.04
*R^2^*	0.23^**^	0.02	0.07
**SPANISH VERSION (*****n*** **=** **466)**			
Psychiatric symptoms	0.50^***^	−0.08	0.09
Time spent online (weekdays)	0.10	0.06	0.20^**^
Time spent online (weekend)	0.09	0.03	0.01
*R^2^*	0.30^***^	0.07	0.12^**^
**TURKISH VERSION (*****n*** **=** **430)**			
Psychiatric symptoms	0.35^***^	0.12	0.02
Time spent online (weekdays)	0.18^*^	0.07	0.17^*^
Time spent online (weekend)	0.08	0.02	−0.10
*R^2^*	0.23^***^	0.03	0.03
**HUNGARIAN VERSION (*****n*** **=** **244)**			
Psychiatric symptoms	0.35^***^	0.01	−0.01
Time spent online (weekdays)	−0.10	0.08	0.00
Time spent online (weekend)	0.32^**^	−0.01	−0.08
*R^2^*	0.36^**^	0.01	0.16
**ENGLISH VERSION (*****n*** **=** **175)**			
Psychiatric symptoms	0.44^***^	−0.15	0.21^*^
Time spent online (weekdays)	0.08	0.00	0.07
Time spent online (weekend)	0.19^*^	−0.04	−0.01
*R^2^*	0.29^***^	0.04	0.07
**GREEK VERSION (*****n*** **=** **154)**			
Psychiatric symptoms	0.52^***^	0.03	−0.25
Time spent online (weekdays)	0.02	−0.03	0.09
Time spent online (weekend)	0.25	0.22	−0.21
*R^2^*	0.43^***^	0.08	0.13

*Gender and age were introduced in the models as control variables. Comparison of β of time spent online (weekdays) and β of time spent online (weekend) (Wald test and p value): Italian sample: 19.5, p < 0.001; German sample: 0.3, p = 0.600; French sample: 8.8, p < 0.01; Polish sample: 3.5, p = 0.0614; Spanish sample: 1.88, p = 0.1705; Turkish sample 0.01, p = 0.997; Hungarian sample: 9.0, p < 0.01; English sample: 2.1, p = 0.1443; Greek sample: 2.9, p < 0.10*.

* p < 0.05;

** p < 0.01;

*** *p < 0.001*.

The influence of psychiatric symptoms and time spent using the internet during weekdays and weekends on the general problem factor and the two specific factors (obsession and neglect + control disorder) was estimated simultaneously via standardized partial regression coefficients in all nine subsamples. Overall, psychiatric symptoms had a moderate-to-strong direct effect on the general problem factor in all subsamples, ranging from β = 0.28 (German subsample) to β = 0.52 (Greek subsample). Results regarding the effect of time spent online during weekdays and at the weekend were not entirely consistent across the subsamples. However, in six out of nine subsamples, results pointed in the same direction. More specifically, beta coefficients were significantly higher for time spent on weekend days than time spent on weekdays in three samples (Italian, French, and Hungarian), higher without reaching statistical significance in another three samples (Polish, English, and Greek), and fairly similar (German and Spanish) or having an opposite direction (Turkish) in the remaining samples, according to the Wald-test. Nevertheless, the degree of the association of time spent online with the general problem factor was low or moderate, or even negative, ranging from −0.14 to 0.32 across the samples. R-squares varied between 13% (German subsample) and 43% (Greek subsample) for the general problem factor.

The effect of psychiatric symptoms on the two specific factors was inconsistent across the nine subsamples. In the majority of the subsamples it was low, non-significant, or even negative. However, in a few subsamples (such as the German, French and Polish subsamples), it was significant with a low effect size. Furthermore, in the majority of the samples, the effect of psychiatric symptoms was fairly similar for the two specific factors. However, in a few subsamples with lower samples sizes (e.g., English and Greek subsamples) the effects were in the opposite direction. The effects of time spent online during weekdays and at the weekend on the two specific factors were low and non-significant in the majority of the subsamples (except for the Spanish and Turkish subsamples, where time spent online during weekdays had a low significant effect on the neglect + control disorder factor). R-squares varied between 0.01 and 0.08 for the obsession specific factor and from 0.03 to 0.16 for the merged specific factor.

## Discussion

The present study tested the psychometric properties of the PIUQ-9 across nine different language-based subsamples and tested four alternative models. These were (i) the original three-factor structure (factors: obsession, neglect, and control disorder), (ii) a two-factor model [factors: obsession and neglect + control disorder (merged due to high correlation among them)], (iii) a bifactor model with a general factor and three specific factors (obsession, neglect and control disorder), and (iv) and another bifactor model with a general factor and two specific factors [obsession and neglect + control disorder (merged due to high correlation among them)]. The results showed that the bifactor model with one general factor and two-specific factors was superior to the other three models and it had an acceptable or good fit in eight out of nine subsamples.

The bifactor model suggests that an overall problem dimension (general problem) exists explaining from 57.0% (German subsample) to 76.5% (Turkish subsample) of the variance in PIUQ-9 across subsamples, while there are two specific factors capturing two qualitatively distinct facets of the problem: obsession and neglect + control disorder. However, according to the results, only the neglect + control disorder factor contained additional information to the general factor, because the proportion of explained variance of the scale score attributed to the obsession factor was low. The neglect + control disorder factor reflects the negative consequences of PIU for the individual's life, being unable to stop using the internet, and neglecting non-internet activities. This is congruent with the DSM-5 formulation of Internet Gaming Disorder and previous descriptions of PIU from a cognitive-behavioral perspective [e.g., (40)]. Furthermore, indicators of dimensionality and reliability, as well as factor loadings, were fairly similar across the nine language-based samples, suggesting some kind of cross-validity of the scale and the proposed bifactor model with two specific factors.

Psychiatric symptoms had moderate-to-strong direct effects on the general problem factor in all subsamples. This is in line with previous findings suggesting psychopathology as measured via the Global Severity Index predicts symptoms of internet addiction (17). Time spent online mostly had low or moderate direct effect on the general problem factor across the nine subsamples. This is also in line with previous findings suggesting that time spent on specific activities (e.g., online gaming, social media use, etc.) in itself is a weak predictor of problematic use (9, 41–43). Overall, looking across all nine samples, the relationship with time spent online appears to be significant in majority of the samples, but lower than in a previous validation study (21). Another interesting finding was that time spent online during the weekend had a significantly higher effect size on the general problem factor than time spent online during weekdays in three of the subsamples, and higher effect size (without reaching statistical significance) in another three subsamples.

Previous studies mostly assessed time spent online with a single question measuring the hours spent online for an entire week or an average day, instead of differentiating between weekdays and the weekends [e.g., (25, 44, 45)]. Nevertheless, it appears that spending time online in these periods of the week is differently related to PIU. Given that the present sample is a general population sample and not a clinical one, this appears reasonable. While treatment-seeking patients in clinical settings often spend most of their time online neglecting work-related and other duties during the week (46), individuals in the general population most likely spend great amounts of time online during the weekend when they have less pressing tasks to do (e.g., household chores rather than tasks related to their occupation and/or education). Therefore, in this population it is reasonable to assume that problems arise from neglecting tasks and hobbies in the individuals' spare time. In most of the subsamples in the present study, PIU was significantly related to internet use on weekend days, while less significant results were found for weekdays. This is in line with previous studies differentiating time spent online during The week and at weekends. Time spent online during both periods was predictive of PIU (47, 48), with higher results for time spent online during weekends and a stronger association with addiction [e.g., (48–50)].

### Limitations

The present study has several limitations that should be noted when interpreting the findings. Causal relationships cannot be established given the cross-sectional design of the present study. The subsamples were self-selected and not representative of the general populations in those countries. Furthermore, sampling methods were slightly different across the nine samples, which may also explain the heterogeneity in terms of age, gender and/or other socio-demographic variables, and is a strong limitation for the replicability of their findings. The data were collected using self-report questionnaires, which are known for producing potential biases (e.g., social desirability biases, short-term recall biases, etc.). Furthermore, the PIUQ-9 was the only measure assessing problematic internet use administered in this study. We decided to limit the number of scales and items as much as possible to increase the likelihood of obtaining larger samples and better quality data. However, the lack of other PIU measures prevented the testing of the incremental validity of the scale. It is also worth noting that the present study used a general population sample, most probably with a low proportion of truly problematic cases concerning internet use. Studies using clinical samples have different results [e.g., (51)]. Finally, the present study was unable provide support for the measurement invariance across different languages in the cross-cultural samples used. This may be explained by the large heterogeneity of samples in terms of gender, age, and other socio-economic variables, as well as daily internet use patterns. Consequently, further studies should use comparable samples to test the measurement invariance of the PIUQ-9.

### Implications

The findings of the present study are important in terms of prevention because they suggest that specific prevention programs should target population groups that show an increased degree of risk related to their sociodemographic, interpersonal (52), and cultural characteristics. The present study using the PIUQ-9 allowed for an understanding of the importance of psychopathological symptoms and time spent online particularly during the weekends in relation with PIU. The relationship between PIU and time spent online in the nine subsamples indicates that stimulus control strategies [e.g., (53)] that limit the person's access to the internet may be useful in dealing with PIU. Moreover, the present study supports that the PIUQ-9 is a short instrument with good psychometric properties across a number of different languages and cultures that can be used in educational and clinical settings as a screening tool to discriminate those at risk of PIU from those not at risk.

## Conclusion

The main objective of the present study was to assess the psychometric properties of the PIUQ-9 across samples from nine different European countries. According to the results, the bifactor model with one general problem factor and two specific factors (i.e., obsession and neglect + control disorder) yielded acceptable or good fit indices in almost all subsamples. Furthermore, psychiatric symptoms had a moderate-to-strong direct effect on the general problem factor in all subsamples, supporting the construct validity of the scale. In a majority of the subsamples, time spent online at the weekend had considerably higher effect sizes on the general problem factor than time spent online during weekdays. The present study highlights the appropriate psychometric properties of the PIUQ-9 across a number of European languages and cultures. Given its brevity, it is especially useful in large-scale studies in which the length of the scale (i.e., survey fatigue) can be an important issue in reducing attrition and increasing the response rate.

## Data Availability

The datasets generated for this study are available on request to the corresponding author.

## Author Contributions

SL, KK-C, DK, AG, IS, AB, FJ, JG, MG-G, NO, KS, GF, and ZD conceived and designed the study. SL, KK-C, DK, AG, IS, AB, FJ, JG, MG-G, NO, KS, GF, ZD, and OK were responsible for data collection. Data analyses were performed by RU and OK with special assistance from SL. OK, RU, MG, and SL wrote the manuscript. All authors provided critical revision to its further development, read and approved the final manuscript.

### Conflict of Interest Statement

The authors declare that the research was conducted in the absence of any commercial or financial relationships that could be construed as a potential conflict of interest.
