# Supplementary material for: Psychometric Evaluation of the Nine-Item Problematic Internet Use Questionnaire (PIUQ-9) in Nine European Samples of Internet Users
Source: Front Psychiatry. 2019 Mar 22;10:136. doi: 10.3389/fpsyt.2019.00136 (PMC6448041; doi:10.3389/fpsyt.2019.00136)
Supplement: Supplementary file 1 [file Table_1.DOCX]

**Appendix. The nine-item version Problematic Internet Use Questionnaire (PIUQ-9) in different languages**

**English PIUQ-9**

In what follows, you will read statements about your Internet use. Please indicate on a scale from 1 to 5 how much these statements apply to you.
1 = never
2 = rarely
3 = sometimes
4 = often
5 = always/almost always

**How often...**

1. do you feel that you should decrease the amount of time spent online?
2. do you neglect household chores to spend more time online?
3. do you feel tense, irritated, or stressed if you cannot use the Internet for as long as you want to?
4. does it happen to you that you wish to decrease the amount of time spent online but you do not succeed?
5. do you spend time online when you’d rather sleep?
6. do you feel tense, irritated, or stressed if you cannot use the Internet for several days?
7. do you try to conceal the amount of time spent online?
8. do people in your life complain about you spending too much time online?
9. does it happen to you that you feel depressed, moody, or nervous when you are not on the Internet and these feelings stop once you are back online?

**Greek PIUQ-9**

Στη συνέχεια, θα διαβάσετε τις δηλώσεις σχετικά με τη χρήση του Internet. Παρακαλείστε να αναφέρετε σε μια κλίμακα 1-5 πόσο αυτές οι δηλώσεις σας χαρακτηρίζουν.
1 = ποτέ
2 = σπανίως
3 = μερικές φορές
4 = συχνά
5 = Πάντα / σχεδόν πάντοτε

πόσο συχνά...

1. Πόσο συχνά αισθάνεστε ότι πρέπει να μειώσετε την ποσότητα του χρόνου που ξοδεύετε στο Διαδίκτυο;
2. Πόσο συχνά παραμελείτε δουλειές του σπιτιού για να περνούν περισσότερο χρόνο στο Διαδίκτυο;
3. Πόσο συχνά νιώθετε ένταση, νεύρα ή πίεση αν δεν μπορείτε να χρησιμοποιήσετε το Διαδίκτυο για όσο χρονικό διάστημα θέλετε να το χρησιμοποιήσετε;
4. Πόσο συχνά σας συμβαίνει να θέλετε να μειώσετε την ποσότητα του χρόνου που ξοδεύετε στο Διαδίκτυο, αλλά δεν τα καταφέρνετε;
5. Πόσο συχνά ξοδεύετε χρόνο στο Διαδίκτυο, ενώ θα προτιμούσατε να κοιμηθείτε;
6. Πόσο συχνά νιώθετε ένταση, νεύρα ή πίεση, αν δεν μπορείτε να χρησιμοποιήσετε το Διαδίκτυο για αρκετές ημέρες;
7. Πόσο συχνά προσπαθείτε να κρύψετε από άλλους το χρόνο που ξοδεύετε στο Διαδίκτυο;
8. Πόσο συχνά οι άνθρωποι στη ζωή σας διαμαρτύρονται ότι ξοδεύετε πάρα πολύ χρόνο στο Διαδίκτυο;
9. Πόσο συχνά σας συμβαίνει να αισθάνεστε θλίψη, αναστάτωση ή νευρικότητα όταν δεν είστε συνδεδεμένοι στο Διαδίκτυο και να σταματήσουν αυτά τα συναισθήματα όταν επιστρέψτε σε σύνδεση;

**Polish PIUQ-9**

Poniżej znajdą się stwierdzenia opisujące sposoby korzystania z Internetu. Prosimy wskazać w skali od 1 do 5, w jakim stopniu te stwierdzenia charakteryzują Pana/Panią.
1 = nigdy
2 = rzadko
3 = czasami
4 = często
5 = zawsze/prawie zawsze

Jak często...

1. ma Pan/Pani poczucie, że powinien/na Pan/Pani ograniczyć czas spędzany online?
2. zaniedbuje Pan/Pani obowiązki domowe, aby spędzić więcej czasu w Internecie?
3. czuje się Pan/Pani spięty/a, rozdrażniony/a lub zestresowany/a, nie mogąc korzystać z Internetu tak długo, jak Pan/Pani chce?
4. zdarzają się Panu/Pani bezskuteczne próby ograniczania ilości czasu spędzanego online?
5. spędza Pan/Pani czas online kosztem snu?
6. czuje się Pan/Pani spięty/a, rozdrażniony/a lub zestresowany/a, nie mogąc korzystać z Internetu przez kilka dni?
7. zdarza się Panu/Pani ukrywać ilość czasu spędzanego online?
8. osoby z Pana/Pani otoczenia narzekają na to, że spędza Pan/Pani zbyt dużo czasu online?
9. zdarza się Panu/Pani odczuwać przygnębienie, zdenerwowanie bądź odczuwać zły nastrój, kiedy jest Pan/Pani poza Internetem, jednak mieć poczucie, że to mija, kiedy jest Pan/Pani ponownie online?

**Spanish PIUQ-9**

A continuación, encontrarás una serie de cuestiones sobre tu uso de Internet. Por favor, indica en una escala de 1 a 5 en qué medida estas cuestiones te caracterizan. 
1 = nunca
2 = raramente
3 = a veces
4 = a menudo
5 = siempre o casi siempre

¿Con qué frecuencia...

1. sientes que deberías disminuir la cantidad de tiempo que pasas conectado?
2. dejaste de hacer tareas en casa para pasar más tiempo conectado?
3. te has sentido tenso, irritado o estresado si no has podido usar Internet tanto tiempo como te gustaría?
4. ha sucedido que deseabas disminuir la cantidad de tiempo que pasas conectado pero no has tenido éxito en conseguirlo?
5. pasas tiempo conectado cuando preferirías dormir?
6. te has sentido tenso, irritado, o estresado si no has podido usar Internet durante varios días?
7. intentas ocultar la cantidad de tiempo que pasas conectado?
8. las personas que te rodean se quejan de que pasas demasiado tiempo conectado?
9. ha sucedido que te has sentido deprimido, de humor cambiante o nervioso cuando no estabas en Internet y esos sentimientos cesaron una vez que pudiste conectarte?

**German PIUQ-9**

Im Folgenden finden Sie Angaben über Ihre Internet-Nutzung zu lesen. Bitte auf einer Skala anzugeben von 1 bis 5, wie viel diese Aussagen kennzeichnen Sie.

1 = nie
2 = selten
3 = manchmal
4 = oft
5 = immer / fast immer

Wie oft...

1. haben Sie das Gefühl, dass Sie die Zeit, die Sie online verbringen, verringern sollten?
2. vernachlässigen Sie Ihren Haushalt, um mehr Zeit online zu verbringen?
3. fühlen Sie sich angespannt, gereizt oder gestresst, wenn Sie das Internet nicht so lange nutzen können wie Sie wollen?
4. passiert es Ihnen, dass Sie die Zeit, die Sie online verbringen, verringern möchten, aber es Ihnen nicht gelingt?
5. verbringen Sie Ihre Zeit online, wenn Sie lieber schlafen würden?
6. fühlen Sie sich angespannt, gereizt oder gestresst, wenn Sie das Internet mehrere Tage nicht nutzen können?
7. verheimlichen Sie wieviel Zeit Sie online verbringen?
8. beschweren sich Menschen in Ihrer Umgebung darüber, dass Sie zu viel Zeit online verbringen?
9. passiert es Ihnen, dass Sie sich depressiv, launisch oder nervös fühlen, wenn Sie nicht im Internet sind und diese Gefühle verschwinden, sobald Sie wieder online sind?

**Italian PIUQ-9**

Di seguito, leggerai delle domande che riguardano come usi Internet. Indica su una scala da 1 a 5 quanto queste affermazioni ti descrivono.
1 = mai
2 = raramente
3 = qualche volta
4 = spesso
5 = sempre/quasi sempre

Quanto...

1. Senti che dovresti ridurre la quantità di tempo trascorsa online?
2. Trascuri le faccende domestiche per trascorrere più tempo online?
3. Ti senti teso, irritato, o stressato se non puoi usare Internet per tutto il tempo che vuoi?
4. Ti succede che vorresti ridurre la quantità di tempo trascorsa online ma non ci riesci?
5. Trascorri del tempo online quando dovresti dormire?
6. Ti senti teso, irritato, o stressato se non puoi usare Internet per alcuni giorni?
7. Cerchi di nascondere il tempo trascorso online?
8. Le persone che frequenti si lamentano che trascorri troppo tempo online?
9. Ti succede di sentirti depresso, lunatico, o nervoso quando non sei su Internet e questi sentimenti spariscono solo se torni online?

**Turkish PIUQ-9**

Aşağıda internet kullanımı ile ilgili durumları okuyarak, sizi tanımlayan durumu 1’den 5’e kadar numaralandırılmış ölçek üzerinde gösteriniz.
1 = asla
2 = nadiren
3 = bazen
4 = sık sık
5 = daima/sürekli

Hangi sıklıkla...

1. internette geçirdiğin süreyi azaltman gerektiğini düşünüyorsun?
2. internette daha fazla vakit geçirmek için evle ilgili işlerini ihmal edersin?
3. İnterneti istediğin kadar kullanamadığın için gerginlik, huzursuzluk ve stres hissedersin?
4. internette geçirdiğin süreyi azaltmayı istediğin halde başaramadığın olur?
5. uyumak yerine internette vakit geçirirsin?
6. İnterneti bir kaç gün kullanamadığın için, gerginlik, huzursuzluk ve stres hissedersin?
7. internette geçirdiğin süreyi gizlemeye çalışırsın?
8. yakınındaki insanlar internette çok fazla vakit geçirmenden şikayet ederler?
9. İnternete girdiğinde geçen ancak İnternete giremediğinde hissettiğin depresif, huysuz ve sinirli olma hallerini hissedersin?

**Hungarian PIUQ-9**

Az alábbiakban az internet-használatoddal kapcsolatos állításokat olvashatsz. Kérjük, jelezd az 1-től 5-ig terjedő skálán, hogy az egyes állítások mennyire jellemzőek Rád!
1 = soha
2 = ritkán
3 = néha
4 = gyakran
5 = mindig/majdnem mindig

Milyen gyakran…

1. érzed úgy, hogy csökkentened kellene az internetezéssel töltött időt?
2. hanyagolod el otthoni teendőidet azért, hogy többet internetezhess?
3. érzed nyugtalannak, feszültnek magad, ha nem internetezhettél annyit, amennyit szerettél volna?
4. van úgy, hogy szeretnéd csökkenteni az internetezéssel töltött időt, de nem sikerül?
5. internetezel olyankor, amikor inkább aludnod kellene?
6. érzed nyugtalannak, feszültnek magad, ha több napig nem tudtál internetezni?
7. próbálod titkolni, hogy mennyi időt töltöttél internetezéssel?
8. panaszkodnak a környezetedben lévők arra, hogy túl sokat internetezel?
9. fordul elő veled, hogy depressziósnak, szomorúnak, idegesnek érzed magad, amikor nem internetezel, és ez az érzés elmúlik, amikor újra internetezni kezdesz?

**French PIUQ-9**

Les questions qui suivent se rapportent à votre **utilisation d’Internet** de manière générale (ni pour le travail, ni pour les études), au cours des six derniers mois. Veuillez cocher les réponses appropriées en utilisant l'échelle suivante : 
1 = Jamais
2 = Rarement
3 = Parfois
4 = Souvent
5 = Toujours/Presque toujours

A quelle fréquence...

1. Devriez-vous diminuer le temps passé sur Internet ?
2. Négligez-vous des tâches quotidiennes pour passer plus de temps sur Internet ?
3. Êtes-vous stressé(e) si vous ne pouvez pas utiliser Internet aussi longtemps que prévu ?
4. Essayez-vous de diminuer le temps passé sur Internet sans y parvenir ?
5. Passez-vous du temps sur Internet alors que vous devriez dormir ?
6. Etes-vous stressé(e) si vous ne pouvez pas utiliser Internet pendant plusieurs jours ?
7. Cachez-vous le temps que vous passez sur Internet ?
8. Les personnes de votre entourage se plaignent parce que vous passez trop de temps sur Internet ?
9. Vous sentez-vous déprimé(e) ou de mauvaise humeur quand vous n’êtes pas sur Internet, et ces sensations cessent dès que vous vous connectez ?
